# Properties of Dry Hopped Dark Beers with High Xanthohumol Content

**DOI:** 10.3390/antiox10050763

**Published:** 2021-05-11

**Authors:** Justyna Paszkot, Joanna Kawa-Rygielska, Mirosław Anioł

**Affiliations:** 1 Department of Fermentation and Cereals Technology, Faculty of Biotechnology and Food Science, Wrocław University of Environmental and Life Sciences, Chełmońskiego 37, 51-630 Wrocław, Poland; joanna.kawa-rygielska@upwr.edu.pl; 2 Department of Chemistry, Faculty of Biotechnology and Food Science, Wrocław University of Environmental and Life Sciences, Norwida 25, 50-375 Wrocław, Poland; miroslaw.aniol@upwr.edu.pl

**Keywords:** fermentation, beer, phenols, antioxidant activity, dark beer, 5-hydroxymethylfurfural, dry hopping, xanthohumol

## Abstract

The antioxidant activity of beers comes mainly from phenolic compounds and melanoidins. The aim of this research was to evaluate the effect of technological operations, especially the ethanol fermentation process using top fermentation brewer’s yeast *Saccharomyces cerevisiae,* on the antioxidant activity of dark dry hopped beers with a high xanthohumol content. Four beers were produced using different varieties of hops. The polyphenol content during beer processing increased at the stage of hopping and fermentation, while it decreased during aging. The ability to reduce iron ions increased for all beers compared to hopped wort. The opposite tendency was noted for the antioxidant capacity expressed as the ability to reduce the radical cation ABTS^•+^ generated from 2,2′-azino-bis (3-ethylbenzthiazoline-6-sulfonic acid). Fermentation and aging caused a decrease in beer color intensity. The content of 5-hydroxymethylfurfural (5-HMF) increased with the color intensity of wort, therefore in beers no presence of 5-HMF was observed. The beers were characterized by a distinctly high content of xanthohumol in the range of 1.77–3.83 mg/L and 0.85–1.19 mg/L of isoxanthohumol. The content of prenylflavonoids and bitterness of beer depended on the variety of hops used.

## 1. Introduction

Beer is an alcoholic beverage obtained by ethanol fermentation of wort consisting of water, malt and hops. In the diet, it can be a source of phytochemicals with positive effects on human health. The bioactive compounds in beer have anti-obesity, anti-cancer, anti-inflammatory, antioxidant and anti-diabetic effects [1]. Regarding the antioxidant activity of beer, it is influenced by the selection of raw materials, the method of brewing wort production, the biological material used, and the parameters of the ethanol fermentation process, as well as the conditions and storage time of the product [2]. 

The antioxidant activity of beer is mainly due to the content of phenolic compounds and melanoidins [3,4]. Phenols in beer are derived from malt (80%) and from hops (20%) [5]. The crucial stages for the extraction of antioxidants in the technological process of beer production are mashing and hopping [6]. The compounds obtained from raw materials during the preparation of brewing wort undergo further transformations with the participation of brewer’s yeast in the process of ethanol fermentation [2]. Beer phenols are classified into the following groups: simple phenols, derivatives of benzoic and cinnamic acids, coumarins, catechins, flavonoids, proanthocyanidins, chalcones, as well as α-acids and iso-α-acids [7]. The presence of polyphenols in beer, apart from a positive effect on antioxidant activity, also results in the appearance of temporary or permanent haze in beer during its storage. This is due to their ability to form complexes with proteins [8]. An interesting compound among beer polyphenols is the hop-derived prenylflavonoid xanthohumol (XN). Its ability to scavenge free radicals is comparable to that of catechins present in green tea, and is several times stronger than the activity of vitamins C and E [9]. Xanthohumol has many beneficial properties, including anti-inflammatory, antiviral, antibacterial, antitumor, antiatherosclerotic, antimutagenic and antioxidant properties [10,11,12]. The only source of this compound in the human diet is beer. However, its content is low, and amounts to about 0.1 mg/L of beer. Dark beers have a higher XN content than pale beers, something which is probably related to the participation of melanoidins formed through the Maillard reaction in the inhibition of the isomerization of XN to a compound with lower biological activity—isoxanthohumol (IXN) [11,12,13,14]. Due to the content of melanoidins and their antioxidant activity, dark beers are also characterized by a higher total content of polyphenolic compounds and antioxidant activity than pale beers [15]. Structurally, they are nitrogenous macromolecular compounds responsible for the brown coloration produced during the process. The content of melanoidins in food cannot be estimated by our limited knowledge of their exact structure. Therefore, the content of the intermediate product 5-hydroxymethylfurfural (5-HMF) can be taken as an indicator of the Maillard reaction [3].

The aim of this research was to evaluate the effect of technological operations and the ethanol fermentation process using top fermentation brewer’s yeast *Saccharomyces cerevisiae* on the antioxidant activity of dark dry hopped beers with high XN content. A unique element of this work is the analysis of the antioxidant properties of four dry hopped dark beers produced with different varieties of hops at subsequent technological stages, with attention to the impact of indicators of the Maillard reaction on wort and beers’ features. We conducted the analysis of total phenolic compounds, antioxidant capacity, 5-hydroxymethylfurfuralcontent, color intensity, glycerol and carbohydrate profiles at successive technological stages with assessment of xanthohumol, iso-α-acids content and the basic physicochemical properties of finished beers. 

## 2. Materials and Methods

### 2.1. Materials and Sample Preparation

#### 2.1.1. Biological Material

Top fermentation brewer’s yeast *Saccharomyces cerevisiae* Safale S-04 (Fermentis, Lesaffre, France) was used in the beer wort fermentation process. Before adding to the wort, the yeast was rehydrated in sterile distilled water at 25 °C for 30 min. In accordance with the manufacturer’s recommendations, a dose of 0.46 g/L was used.

#### 2.1.2. Raw Materials

The following malts were used in the production of beer wort: Pilsner malt (3–4.5 EBC, Viking Malt; Strzegom, Poland) and dark chocolate malt (1100–1300 EBC, Viking Malt; Strzegom, Poland). The beers were hopped with the hop varieties Marynka (Twój Browar, Poland, 8.8% α-acids), Amarillo (Twój Browar, Poland, 8.8% α-acids), Cascade (Twój Browar, Poland, 7.7% α-acids), Centennial (Twój Browar, Poland, 8.5% α-acids) and Galaxy (Twój Browar, Poland, 13.3% α-acids).

#### 2.1.3. Brewing Technology

The wort was obtained by filtering the mash, which was produced using the two-step decoction method. The mash was made with 90% Pilsner malt and 10% dark chocolate malt. The mashing process steps were as follows: 52 °C for 10 min, 63 °C for 40 min, 72 °C for 30 min and 78 °C for 10 min. The temperature increase was obtained by taking 1/3 of the mash volume, boiling in a separate vessel for 10 min, and returning it to the main mash tun. After mashing, the mash was filtered. The obtained wort (10 L) was boiled with the addition of Marynka hop (12 g). The wort extract was then set at 12 °Bx by measuring with a Densito 30PX densimeter (Mettler Toledo, Columbus, OH, USA) (Analitica EBC, 2010). The wort was divided into four samples (2 L volume). Then, separately, the samples with the addition 2.5 g/L of hops (samples: A—Amarillo, B—Cascade, C—Centennial, D—Galaxy) were boiled for 10 min. The worts were cooled down to 18 °C and fermented with *S. cerevisiae* Safale US-04 top fermenting yeast (Fermentis, Lesaffre, France) at a dose according to the manufacturer’s recommendations (0.46 g/L) and temperature of 18 °C. Main fermentation was carried out for seven days. The beers were then decanted from the yeast sediment into disinfected fermentation flasks. The next stage of the technological process was dry hopping of the beers using a dose of 5 g of one of the hop varieties (samples: A—Amarillo, B—Cascade, C—Centennial, D—Galaxy). The beers were post-fermented for seven days at 18 °C, then poured into 500 mL bottles. Carbonation was obtained as a result of refermentation. For this purpose, sucrose was added to the beer in a dose of 4.7 g/L before bottling. The beers were aged for 4 weeks at 4 °C.

#### 2.1.4. Sample Preparation and Abbreviations

The beers were degassed in a 358A laboratory shaker (ElpinPlus, Lubawa, Poland) and filtered into Erlenmeyer flasks through a pleated filter. Before analysis, both the wort and beers were centrifuged in a laboratory centrifuge (MPW-351R type) in Falcon 50 cm^3^ containers (5000 rpm for 10 min). The obtained samples were decanted.

The following abbreviations were used to mark the samples: W0—wort after filtration, W1—wort hopped with bitter hops (Marynka), A—wort and beers hopped with the Amarillo variety, B—wort and beers hopped with the Cascade variety, C—wort and beers hopped with the Centennial variety, D—wort and beers hopped with the Galaxy variety. Technological stages have been additionally marked with numbers: 1—hopped wort, 2—beer after main fermentation, 3—beer after post-fermentation and 4—beer after maturation.

### 2.2. Analytic methods

#### 2.2.1. Basic Physico-Chemical Parameters

The beers were degassed using a 358 A laboratory shaker (Elpin Plus, Lubawa, Poland), and then filtered using paper filters and diatomaceous earth. Next, using a beer densimeter Anton Paar beer analyzer DMA 4500 M (Graz, Austria), the ethanol content, real attenuation, apparent attenuation, wort extract content, color (EBC) and beer density were measured.

#### 2.2.2. Determination of Beer Bitterness (IBU)

The analysis of iso-α-acids content was performed according to Analytica EBC [16]. A sample of degassed beer (10 cm^3^) was transferred to a 35 cm^3^ Falcon tube. Then 6 N HCl solution (0.5 cm^3^) and isooctane (20 cm^3^) were added. The samples were shaken for 5 min, before 10 cm^3^ of the mixture was transferred to a 15 cm^3^ Falcon centrifuge tube. The sample was centrifuged (2675 centrifugal force (RCF)) in Falcon containers (5 min, 3000 rpm). Then a sample was taken from the isooctane layer and determined with a BECKMAN DU-650 UV-2401 PC spectrophotometer (Shimadzu, Kyoto, Japan) at a wavelength of 275 nm. Pure isooctane was the reference sample. The content of iso-α-acids was calculated according to the formula: IBU= 50A (IBU—brewery units [mg/dm^3^], A—absorbance at 275 nm).

#### 2.2.3. High-Performance Liquid Chromatography (HPLC) Analysis of Carbohydrates Content

Carbohydrate profiles (maltose, maltotriose, glucose, dextrin) and glycerol content were analyzed by high-performance liquid chromatography (HPLC) [17]. Samples were degassed and centrifuged with a laboratory centrifuge MPW-351R (10 min, 5000 rpm). Then the samples were diluted with water and filtered through 0.22 µm filters. A 5-fold dilution for wort and a 2-fold dilution for beer samples was used. Separation was made using Rezex ROA—Organic Acid H+ (300 × 7.8 mm) column (Phenomenex, Torrance, USA). The HPLC method, using Prominence (Shimadzu, Kyoto, Japan) was used. Measurement parameters: injection volume—0.02 mL, flow rate—0.6 mL/min, temperature of separation—60 °C, mobile phase—0.005 mol/dm^3^ H_2_SO_4._ A refractometric method of detection was used. All measurements were performed in triplicate. Results were presented as g/L of wort or beer.

#### 2.2.4. High-Performance Liquid Chromatography (HPLC) Analysis of Xanthohumol (XN) and Isoxanthohumol (IXN) Content

The content of XN and IXN was analyzed using the method by Jurková et al. [18] with modifications. The analysis consisted of solid-phase extraction (SPE) using the Strata C18-E, 500 mg/6 mL column (Phenomenex, Torrance, CA, USA). For the analytes separation from beer samples, we used: No.1—methanol, No. 2—water:H_3_PO_4_ (100:0.2 *v/v*), No. 3—methanol:water:H_3_PO_4_ (20:80:0.2 *v/v/v*) and No.4—methanol:H_3_PO_4_ (100:0.2 *v/v*). Columns were conditioned by passing 10 mL of No. 1, and next 10 mL of No. 2. The acidified beer samples (25 mL) were applied to the SPE columns, treated with 10 mL of No. 3 and allowed to “dry” for 10 min under vacuum. The analytes were washed out with 5 mL of No. 4 solution into 2.5 mL measuring tubes to the level just before the mark, filled with the same solution and mixed. Samples were filtered through a 0.45 Pm PTFE filter and analyzed by HPLC.

High-performance liquid chromatograph (Waters 2690) with a diode array detector (Waters 996) was used. The analyzes were performed on a C-18 reverse phase column (Kinetex 5u XB-C18 100A). The column was thermostated at 28 °C and the analyzed samples at 12 °C. The following solutions were used as eluent: A- 1% vol. formic acid in water; B-1% vol. formic acid in acetonitrile. Elution program: 65% A-10 min., 65% A-10% A-8 min., 10% A-4 min., 10% A-65% A-1 min., 65% A-7 min. The flow rate was 1.5 mL/min. The XN and IXN external standard (0.535 mg/mL of XN and 0.496 mg/mL of IXN) was used in the analysis. The analyses were performed in triplicate. Results were presented in mg of XN or IXN per liter of beer.

#### 2.2.5. High-Performance Liquid Chromatography (HPLC) Analysis of 5—Hydroxymethylfurfural (5-HMF) Content

The content of 5-HMF in wort and beers was analyzed by high-performance liquid chromatography using the Dionex system (Thermo Fisher Scientific, Bremen, Germany) and the TCC-3000SD Cadenza Imtakt CD-C18 column (75 × 4.6 mm, 5 µm) [17]. An UltiMate 3000 diode detector, LPG-3400A pump and EWPS-300SI autosampler were used. The mobile phase was composed of solvents A (4.5% *v/v* formic acid) and B (100% acetonitrile). An injection volume of 20 µL and an elution rate of 1.0 mL/min were used. The elution procedure was as follows: 0–1 min 5% B in A, 20 min 25% B in A, 21 min 100% B, 26 min 100% B, 27 min 5% B in A. The column temperature was 30 °C. 5-HMF was identified at 280 nm. Chromeleon v.7.2.—Chromatography Data System (Thermo Scientific Dionex, Sunnyvale, CA, USA) was used for integration and quantification of the data. Results were presented as mg/L of wort or beer.

#### 2.2.6. Total Polyphenols Content

The Folin–Ciocalteu (F–C) spectrophotometric method was used to determine the total polyphenol content (TPC) in worts and beers [19]. The analyzed wort or beer samples (0.1 mL) and F-C reagent (0.2 mL) were mixed in plastic cuvettes and incubated for 3 min. A 20% aqueous sodium carbonate solution (1 mL) and distilled water (2 mL) were then added to the samples. The blank was distilled water. After one hour of incubation, the absorbance was analyzed spectrophotometrically using UV-2401 PC (Shimadzu, Kyoto, Japan) at a wavelength of 765 nm. The results are presented as the mean value of triplicates. A calibration curve in the range of 0.30–9.00 mg GAE/L was used to read the results.

#### 2.2.7. Ability of Iron Ion Reduction (FRAP)

An analysis of the ability to reduce ferric ions was performed using the method by Benzie and Strain [20]. The FRAP (ferric reducing antioxidant power) reagent was prepared by combining 20 mL of an aqueous solution of iron (III) chloride (0.1018 g FeCl_3_) with a solution of 2,4,6-Tris(2-pyridyl)-s-triazine (0.0664 g TPTZ) in 40 mM hydrochloric acid (20 mL HCl) with acetate buffer pH 3.6. Quantitative analysis was performed by the external standard method using iron (II) sulfate (0.2 mmol/L) (VI) as reference. A correlation curve between the absorbance value and the compound concentration was prepared. Beer (1 mL) diluted in distilled water and FRAP reagent (3 mL) were mixed in cuvettes. Distilled water was used as a blank. The absorbance was determined at fli 593 nm with a UV-2401 PC spectrophotometer (Shimadzu, Kyoto, Japan). The results are presented as the mean of triplicates in millimoles of Trolox per liter of wort or beer.

#### 2.2.8. Ability of Radical Cation ABTS^•+^ Reduction

Antioxidant activity was determined using the radical cation reduction method ABTS^•+^ [21]. The wort or beer samples (0.03 mL) were mixed with the 2,2′-azino-bis (3-ethylbenzthiazoline-6-sulfonic acid) (ABTS^•+^) solution with a determined absorbance value (0.700). After 6 min of incubation, the measurement was made using UV–2401 PC spectrophotometer (Shimadzu, Kyoto, Japan) with the wavelength 734 nm. Determinations were performed in triplicate. Results were presented in mmol of Trolox per liter of wort or beer.

#### 2.2.9. Statistical Analysis

One-way analysis of variability (ANOVA) was performed for the obtained data. The significance of differences between the mean values was tested using Duncan’s test (*p* < 0.05). Statistical analysis was performed using the Statistica 13.5 program (StatSoft, Tulsa, OK, USA).

## 3. Results and Discussion

### 3.1. Physicochemical Properties of Beers

Finished beers, after the aging process, were analyzed with the use of an oscillating densimeter with a beer analyzer (Table 1). The beers did not differ significantly in ethanol content (5.37—5.49% *v/v*). The values of apparent and real attenuation differed statistically significantly, but the magnitude of these differences was not large. The apparent attenuation ranged from 80.27 to 81.33% *w/w*, while the real attenuation 64.93 to 65.73% *w/w*. Similar parameters (ethanol content, attenuation and final beer density) resulted from the use of wort for fermentation with similar extract content (12.31–12.61% *w/w*). The above-mentioned parameters allowed us to conclude that the variety of hops used did not have a significant impact on the process of fermentation of beer wort.

The addition of dark malt did not significantly affect the apparent extract of wort, while the samples were clearly differentiated in terms of the real extract, the degree of attenuation, and the final ethanol content in the beer. Although wort had a similar extract content, the attenuation level and ethanol content were lower for beers with a higher proportion of dark malt, while the real extract content and density increased. This may indicate the fact that dark wort contains sugars that are less susceptible to fermentation, or that the Maillard substances inhibit yeast metabolism. Research by Coghe et al. [22] gave very similar results to those obtained in our experiment. They also used 10% dark malt to prepare the wort, and the apparent extract of the beers was (as in our trial) close to 12 °Plato. The beers they obtained were characterized by a lower real extract and a higher degree of attenuation than our beers. These values confirm that there are less fermentable sugars in darker-colored malt. The ethanol content in the beers obtained in both experiments is at a similar level (about 5.3% *v/v*). The higher EBC value of the malt used in our research did not significantly affect the alcohol content of the produced beers [22].

Hopped wort differed statistically significantly in terms of the content of all tested sugars (Table 2). Neither of them contained glycerol. Maltotriose, maltose, and glucose contribute to the ethanol fermentation process with the use of *S. cerevisie* brewer’s yeast, therefore their content decreases during the process. Maltose is the main sugar of wort [23]. The wort contained 49.21–58.02 g/L maltose, 12.06–15.13 g/L maltotriose, 6.53–8.34 g/L glucose and 30.68–46.85 g/L dextrin. The D1 wort was characterized by the highest content of all tested sugars. After the fermentation process, the content of maltose decreased significantly to 0.34–0.36 g/L. There were no statistically significant differences in the content of this sugar in beers after the main fermentation, post-fermentation, and maturation. After main fermentation and post-fermentation, no glucose content was noted in any of the tested beer variants. Its small amounts were contained in the sample of A4 beer at the final stage of the technological process—after maturation. This may be due to the addition of glucose to the beer prior to bottling as a raw material for refermentation, which is used to naturally saturate beer with carbon dioxide.

The maltotriose content in beers after main fermentation (4.51–5.31 g/L) results from the order of use carbohydrates by the brewer’s yeast. First, *S. cerevisiae* yeast metabolizes monosaccharides (glucose and fructose), then disaccharides (maltose and sucrose), and finally maltotriose [24]. Dextrins are carbohydrates that do not undergo ethanol fermentation using *S. cerevisiae* brewer’s yeast, and therefore the significant changes in their content observed in the trials cannot be related to the use of these carbohydrates in the fermentation process. The content of dextrin decreased significantly after fermentation for trials B3 and C3, and after aging compared to beers after the main fermentation for trials A4, B4, C4. The decrease in dextrin content during the process of dry hopping is attributed to their hydrolysis with the participation of enzymes present in hops added to cold beer or enzymes of microbiological origin. The presence of the following enzymes in hops has been documented: amyloglucosidase, α-amylase, β-amylase, and limit dextrinase. It is worth noting that the material tested in that experiment was Cascade hop, which was also used in our experiment (variant B). The activity of hop enzymes is the cause of fermentable sugars appearing in beer after dry hopping and a decrease in the content of dextrin. Their action may increase the attenuation of beer. Thanks to the action of enzymes from hops, an additional dose of sugars appears in the beer, which causes the ethanol fermentation to reinitiate. This phenomenon is called ‘hop creep’ [25]. The reason for resuming fermentation after dry hopping may also be the fact that a certain dose of sugar is introduced into the beer with hops. About 2% of the dry weight of hop cones are monosaccharides [5].

Glycerol is synthesized by *S. cerevisiae* yeast cells. It is responsible for protecting the cell against osmotic and thermal stress, and also affects the sensory features of beer defined as body and fullness [26]. The glycerol content changed with the successive stages of the technological process. It is the main byproduct of ethanol fermentation, which explains its lack in brewing wort. In finished beers, its content ranged from 2.74–3.03 g/L. Han et al. [27] examined 34 pale beers for glycerol content. The beers they tested had from 925.2 to 1.502.74 mg/L. Glycerol biosynthesis by *S. cerevisiae* depends on the temperature of the fermentation process, the strain of brewer’s yeast, sugar content in the fermenting wort, amount and type of nitrogen source, pH value, SO_2_ and oxygen content in the culture medium [26].

The bitterness of the beers was also analyzed. The beers were characterized by the bitterness level index in the range of 55.45–75.22 IBU. This indicator is related to the content of iso-α-acids in beer. In the experiment, Marynka bitter hops were used—the same for all produced variants of beers. The samples were differentiated in terms of the variety of aromatic hops used, which, to a lesser degree, affect the bitterness of beer. The varieties of hops used differed in the content of α-acids. They contained from 7.7–13.3% of these bitter compounds. The determined value of bitterness in beer depends on the content of these compounds in the type of hops used.

### 3.2. The Content of Xanthohumol and Isoxanthohumol

Hops are responsible for the beer’s characteristic aroma and bitter taste. It is also a source of several bioactive substances. One group of compounds found in hops with a wide range of health-promoting properties are flavonoids. Due to the presence of hydroxyl groups and multiple bonds in the structure of these compounds, they have the ability to protect the body’s cells against reactive oxygen species. A group of flavonoids with one or more prenyl groups is called prenylated flavonoids. As much as 95% of the prenylated hop flavonoids are prenylated chalcones. Among them, the dominant is xanthohumol, constituting 80–90% of the prenylflavonoids. In a smaller amount than XN, another prenylated flavonoid, desmethylxanthohumol, is also present in the lupulin glands of hop cones. As a result of thermal isomerization in beer wort, this compound is transformed into a racemic mixture of enantiomers 6-prenylnaringenin and 8-prenylnaringenin, which is the most potent plant phytoestrogen known [5].

The results of the XN and IXN content in obtained beers are presented in Table 3. The samples differed significantly in terms of the content of both prenylflavonoids. The beers were characterized by a high XN content, in the range of 1.77–3.83 mg/L, while IXN was between 0.85–1.19 mg/L. The highest XN content was found in D4 beer, hopped with the Galaxy hop variety, while the lowest in B4 beer, hopped with the same dose of Cascade hop. Compared to the XN content in beers tested by other authors, our results are outstanding. This is due to the specially designed beer production technology. The use of decoction mashing allowed efficient extraction of colored compounds from malt. As the amount of melanoidin in the wort increases, the loss of XN during the boiling of the wort decreases. This indicates the participation of these compounds in inhibiting the thermal isomerization of chalcones to flavanones [13]. Additionally, in our experiment application of the dry hopping process, in which the thermal factor favoring isomerization of XN is excluded, we were able to obtain a high content of prenyloflavonoids in the final beer. Stevens et al. [28] analyzed 13 beers for the content of xanthohumol, isoxanthohumol, 6-prenylnaringenin, and 8-prenylnaringenin. The research showed that the total content of these four compounds in beer was in the range of 0–4 mg/L. Lager-type beers contained 0.009–0.034 mg/L of XN, while 0.4–0.68 mg/L of its isomerization product was IXN. Stout and porter beers had a higher XN content from pale beers (0.34 mg/L and 0.69 mg/L, respectively). The study of the XN content in 27 Polish beers was undertaken by our department. In these beers, the XN content was determined to be in the range of 0.006–0.22 mg/L, and the highest results were also recorded for dark beers and unfiltered light beers with extract content greater than 12.5% *w/w* [29]. The higher XN content in dark beers is probably related to the presence of melanoidins, which are high molecular weight end products of the Maillard reaction [13].

Xanthohumol is the dominant substance in the composition of β-resins in hops. Its content in hop cones is 0.3–1.5% of dry matter. It is a prenylated chalcone present in the lupulin glands of hops. Although XN is the main component of the hard resins of hops, only a small amount of it passes into the beer as a result of the traditional production process. It has been estimated that it goes into the finished product with a yield of only 5%. This is due to its hydrophobic nature, which makes it poorly soluble in wort. Large amounts of XN are removed from the wort as the hops separate and lost during fermentation. Part of it is absorbed by yeast cells and separated by clarification processes using PVPP (polyvinylpolypyrrolidone). As a result, the content of XN in the finished beer is usually lower than 0.1 mg/L [13].

Isoxanthohumol is a compound with better solubility in wort than xanthohumol, but shows lower pro-health activity. However, there are studies showing its activity as a phytoestrogen due to its conversion into 8-prenylnaringenin [30]. It was shown that the content of IXN in commercial beers ranges between 0.04–3.44 mg/L in alcoholic beers and reaches about 0.11 mg/L in nonalcoholic beers. The results of IXN content obtained in our experiment are similar to Stevens et al. [28].

Researchers have repeatedly attempted to enrich beer in XN using an addition of dark malt, modified hopping technologies, using preparations rich in XN dosed at different stages of beer production, skip filtering and stabilizing the beer with PVPP or activated carbon, reuse of yeast biomass for fermentation, and changes in pH of the wort before hopping [11,13]. Wunderlich et al. [13] obtained beer with an XN content of 17.2 mg/L as a result of using an 80 mg/L dose of XN and 10% dark malt. In our experiment, we obtained beer using conventional hopping without the use of XN extracts. It is also worth noting that our results are close to the content of XN showing biological activity (5 mg/L) [31].

### 3.3. The Content of 5-Hydroxymethylfurfural and the Color (EBC) of Wort and Beers

The color of the beers depends mainly on the malts used in the wort production process. During the production of beer, it undergoes little change, mainly at the stage where the wort is subjected to thermal treatment. The color of the wort (Table 4), before boiling with hops, was 106.73 EBC. The process of hopping with bitter hops led to a significant darkening of wort (to 133.43 EBC). At the same time, the content of 5-HMF, one of the markers of the Maillard reaction, increased. For W0, it was 18.92 mg/L. The content of 5-HMF in brewing wort produced with the participation of 10% of various types of dark malt ranges from 5.54–24.31 mg/L, assuming the highest value for the wort produced with dark chocolate malt [23]. The wort, before hopping W0 obtained in the experiment, has a lower 5-HMF content. After boiling with bitter hops, the 5-HMF level rose to 24.65 mg/L (W1), which was due to the Maillard reaction changing during heating.

After the next technological stage, boiling with aromatic hops, the samples were characterized by a color of between 114.15 to 142.95 EBC and a content of 5-HMF at a level of 20.04 to 26.72 mg/L. The 5-HMF content increases with increasing EBC colour index. The intensity of boiling of the trials during hopping may be the reason for the differences in the results between the variants, differing in the type of hops used in the production process. As a result of the main fermentation, the color intensity of the beers decreased. The decrease ranged from 6.2% (A2) to 19.0% (B2). This may be the result of melanoidin decolorization by the enzyme manganese peroxidase from yeast *S. cerevisiae*. Kahraman et al. [32] showed that *S. cerevisiae* yeast caused a 33% decolorization of molasses wastewater, which were a byproduct from the sugar industry. In turn, Tsiakiri et al. [33] showed that the immobilized *S. cerevisiae* baker’s yeast is capable of 80 to 100% reduction of melanoidin from solutions of various initial concentrations after 48 h of incubation. The reduction in the content of colored compounds is due to the activity of laccase and peroxidase stimulated by Cu^2+^ and Mn^2+^ ions.

After post-fermentation and maturation, the color of the beers also changed, but on a smaller scale compared to the earlier technological stage. 5-HMF is one of the intermediates in the Maillard reaction. The fermentation process carried out with the participation of *S. cerevisiae* depends on the presence of 5-HMF. The effect of doses of 0.5–15 g/L of 5-HMF was analyzed by Da Silva et al. [34]. The results indicate that the content of 1–10 g/L in the medium prolongs the yeast lag phase period proportionally to the dose, while with the use of 15 g/L the growth of yeast is completely inhibited. 5-HMF is the product of a series of reactions that occur as a result of heating a medium containing pentosis and hexosis. It hinds the fermentation process by inhibiting enzymes from the group of dehydrogenases involved in the metabolism of *S. cerevisiae* [35].

After the ethanol fermentation process, a complete reduction of the 5-HMF content was observed in all tested beer variants. 5-HMF is converted to 5-hydroxymethylfurfuryl alcohol (5-HMF alcohol) during ethanol fermentation. The yeast *S. cerevisiae* has been shown to process 5-HMF into 5-HMF alcohol both under aerobic and anaerobic conditions [36]. Viegas et al. [37], analyzed commercial beers in various styles for the content of 5-HMF. They showed that dark beers contain an average of 6.99 mg/L 5-HMF, amber beers 6.84 mg/L, while light beers contained 4.29 mg/L. The beers in our analysis were tested shortly after the finished production process, while commercial beers tested by Viegas et al. [37] were obtained from supermarkets. The higher content of 5-HMF may be due to its formation during the storage of beers.

### 3.4. Total Polyphenol Content (TPC) and Antioxidant Activity

The total content of polyphenols (TPC) and the antioxidant activity of wort and beers at subsequent production stages are presented in Table 5. The content of polyphenols in the wort (W0) was 336.13 mg GAE/L. The ability to reduce iron ions (FRAP) and the antioxidant capacity (ABTS^•+^) wort were 1.61 and 0.91 mmol TE/L, respectively. After hopping the wort with bitter hops (W1), the TPC value rose to 388.19 mg GAE/L (an increase of 15.5% over the wort before hopping). The antioxidant potential ABTS^•+^ of wort also increased by 39.5%, while FRAP by 20.8%.

TPC in hopped worts increased for samples A1 and D1 by 7.7% and 22.9%, respectively. The sample hopped with the Galaxy (D1) variety was characterized by the highest content of polyphenolic compounds. The lowest content was found in samples B1 and C1, where TPC slightly decreased in relation to W1. After hopping, ABTS^•+^ and FRAP antioxidant potential increased (by 19.5–43.0% for ABTS^•+^ and 20.3–43.0% for FRAP results). Hop varieties differ in content of phenolic compounds. Their quantitative and qualitative composition in hops is also determined by the harvest date, place and climatic conditions of cultivation as well as the method of storage [38].

The phenolic compounds undergo changes during the thermal treatment. Such processes may occur during the boiling of the beer wort. For some of the polyphenols, heat treatment is associated with an increase in antioxidant activity. Moreover, the antioxidant ability is a feature more resistant to high temperatures than the content of polyphenols. The presence of other compounds in the environment influences the behavior of polyphenols under high temperature conditions [39].

All variants after the main fermentation had a higher TPC than before the process. There was an increase in the parameter from 30.8% for D2, up to even 86.0% for C2. The sample C2 contained 713.8 mg GAE/L, while D2 623.91 mg GAE/L. The FRAP assay results did not change statistically after fermentation for samples D2 and B2. For the remaining samples, we noted a slight increase in the FRAP capacity. Differently in the case of the radical reduction potential (ABTS^•+^). A statistically significant decrease in antioxidant power was noted for B2 (by 24.5%) and C2 (by 18.45%). The increase in antioxidant capacity after ethanol fermentation may be a result of the overproduction of NADH by yeast in the process of glycolysis. It is presumed that the conversion of sugars to pyruvate or acetaldehyde during ethanolic fermentation is faster than acetaldehyde to ethanol. It results in increased NADH (nicotinamide adenine dinucleotide) production, and this in turn leads to an increase in the antioxidant potential of beers after ethanol fermentation [40].

After post-fermentation, an increase in TPC was observed in trials B3, C3, and D3. There were no statistically significant changes in the TPC content in sample A3 in relation to A2. At this stage, the C3 had the highest TPC content (840.75 mg GAE/L), while A3 had the lowest (693.34 mg GAE/L). After post-fermentation, the increase in polyphenol content was not as high as after the main fermentation. There was an increase in TPC from 2.5% (A3) to 17.8% (C3). It is worth noting that after separating the beer from the yeast sediment after the main fermentation, the beers were dry hopped. Dry hopping increases TPC by up to 49% [41], so it could be compensated for the decrease in TPC during post-fermentation. The ABTS^•+^ antioxidant activity changed significantly only for D3, the remaining trials after fermentation showed no statistically significant changes in this parameter. This is not the case for FRAP activity. For this method, post-fermentation and dry hopping led to significant changes. There was a decrease in FRAP antioxidant activity for A3 and C3 (8.1% and 17.1%), while for B3 and D3 an increase (9.0% and 10.8%).

After bottling and maturation, the beers showed a statistically significant decrease in the content of TPC, regardless of the variant, from 35.1% (D4) to even 55.8% (C4). The finished beer with the highest TPC was D4 (475.05 mg GAE/L). The obtained results are not consistent with Pascoe et al. [1]. They observed an increase in antioxidant activity as a result of the mashing process, after filtration, hopping, as well as post-fermentation and pasteurization. Moreover, after filtration, storage, and bottling of beer, this increase was correlated with an increase in the content of the most monitored polyphenolic compounds [1]. Our experiment confirms that the content of polyphenols rises at the stage of main fermentation and post-fermentation but significantly decreases during maturation. McMurrough et al. [42] described the transformation of beer flavanols during storage. In 4–5 weeks, they noted a large loss of tested compounds. Monomeric and dimmeric flavanols disappeared, while the content of macromolecular compounds (tannins) increased. These processes can explain the reduction in TPC in the tested samples after maturation. It may also be caused by the spontaneous sorption of polyphenols by sediments and the polymerization of catechin and epicatechin [43]. Polyphenolic compounds also contribute to the formation of haze in beer. Polymerized to form macromolecular compounds forming sediments, which may also be the cause of a decrease in TPC [44].

Zhao et al. [45] showed that lager beers had a lower TPC content that beers obtained by us. It ranged from 152.01 mg GAE/L to 339.12 mg GAE/L. In Canadian beers, the content of polyphenols was between 3.72–13.73 mg GAE/100 mL, with the highest value for dark beer seen in stout style [45]. Phenolic compounds are responsible for the antioxidant activity in 55.0–88.1%. The most abundant polyphenolic in beer samples are gallic acid and ferulic acid. In turn, Piazzon et al. [46] tested commercial beers in the following styles: lager, pilsner, wheat, ale, abbey, and bock. The bock-style beer was characterized by the highest TPC determined by the Folin–Ciocalteu method (875 mg GAE/L). This value is almost twice as high as that obtained for the beer with the highest polyphenol content in our experiment. Abbey and wheat beers also had higher TPC values, while lager and pilsner styles are similar to the beers we obtained. Piazzon et al. [46] analyzed the beers in terms of FRAP antioxidant activity. We found that that despite the much lower content of polyphenolic compounds, the antioxidant activity of the beers produced in our experiment is similar to bock beer (4.7 µmol TE/mL). The remaining tested beers styles show clearly lower activity (2.2–3.6 µmol TE/mL) [46].

According to Pulido et al. [47], the content of polyphenols in dark beer ranges from 37.3–57.2 mg GAE/100 mL. These results were similar to those obtained in our experiment. Compared with lagers, dark beers have a much higher polyphenol content. Pale beers contain about 31.2-37.0 mg GAE/100mL. It is worth noting that the dark beers we obtained had an antioxidant activity higher than that of white wine (1.54 µmol T/mL) and rose wine (2.86 µmol T/mL), as well as orange juice (5.15 µmol T/mL), while red wine, coffee and tea are significantly stronger as antioxidants.

## 4. Conclusions

The ethanol fermentation process, with the use of *Saccharomyces cerevisiae* top-fermented yeast, led to an increase in total polyphenol content for each tested sample when compared to responding hopped worts. The content of polyphenols increased after the main fermentation and post-fermentation, while it decreased after the maturation process. The ferric ion reduction ability (FRAP) rose at all process stages up to post-fermentation, where little variation was noted depending on the variant. After maturation, it increased again and achieved the highest value in the whole process. In turn, the ability to scavenge ABTS^•+^ free radicals increased during hopping, while it decreased after maturation. The use of different varieties of aromatic hops led to the obtaining of beers diversified in terms of total polyphenol content, antioxidant potential, while to a lesser extent influencing the beer parameters such as: ethanol content, attenuation level or extract, as well as the carbohydrate profile and glycerol content in beers. The ethanol fermentation process led to a complete reduction of the 5-hydroxymethylfurural content and to significant changes in the color of the beers. The beers were characterized by a distinctly higher xanthohumol content compared to commercial beers, or beers produced using conventional methods without enrichment with preparations rich in this prenylated flavonoid. The analysis of changes in sugar content during the production process allowed to observe the effect of dry hopping on changes in the carbohydrate profile, probably resulting from the activity of enzymes present in the hops.

## Figures and Tables

**Table 1:** Physicochemical properties of beers.

Sample	Ethanol	Attenuation		Extract		Density	Bitterness
		Apparent	Real	Apparent	Real		
	% *v/v*	% *w/w*		% *w/w*		g/cm^3^	IBU
A4	5.42 ± 0.03 ^a^	80.71 ± 0.02 ^c^	65.21 ± 0.02 ^c^	12.61 ± 0.05 ^a^	4.39 ± 0.02 ^a^	1.0076 ± 0.00 ^b^	59.45 ± 0.10 ^b^
B4	5.37 ± 0.05 ^a^	81.33 ± 0.01 ^b^	65.67 ± 0.01 ^b^	12.31 ± 0.03 ^c^	4.23 ± 0.02 ^c^	1.0072 ± 0.00 ^d^	55.45 ± 0.67 ^c^
C4	5.42 ± 0.02 ^a^	80.385 ± 0.02 ^a^	65.73 ± 0.02 ^a^	12.43 ± 0.02 ^b^	4.33 ± 0.01 ^b^	1.0075 ± 0.00 ^c^	56.35 ± 0.31 ^c^
D4	5.49 ± 0.02 ^a^	80.27 ± 0.02 ^d^	64.93 ± 0.02 ^d^	12.60 ± 0.02 ^a^	4.4 ± 0.01 ^a^	1.0078 ± 0.00 ^a^	75.22 ± 1.16 ^a^

Results are presented as a mean of three replicates ± standard deviation. The mean values presented in one column, marked with different letters (a, b, c, etc.), differ statistically (*p* < 0.05).

**Table 2:** Carbohydrate profiles and glycerol content in hopped worts and beers.

Sample	Maltose	Maltotriose	Glucose	Dextrins	Glycerol
g/L					
Hopping					
A1	52.59 ± 0.23 ^c^	13.63 ± 0.10 ^b^	7.53 ± 0.20 ^b^	39.46 ± 0.08 ^d^	nd
B1	49.21 ± 1.6 ^d^	12.13 ± 0.05 ^c^	7.01 ± 0.05 ^c^	34.46 ± 0.35 ^g^	nd
C1	53.87 ± 0.79 ^b^	12.06 ± 0.45 ^c^	6.53 ± 0.15 ^d^	30.68 ± 0.56 ^h^	nd
D1	58.02 ± 0.90 ^a^	15.13 ± 0.26 ^a^	8.34 ± 0.39 ^a^	46.85 ± 1.32 ^a^	nd
**Main fermentation**					
A2	0.36 ± 0.01 ^e^	5.17± 0.10 ^d^	nd	38.49 ± 0.78 ^de^	3.39 ± 0.03 ^a^
B2	0.34 ± 0.00 ^e^	4.83 ± 0.06 ^e^	nd	37.28 ± 0.34 ^ef^	2.61± 0.03 ^g^
C2	0.36 ± 0.00 ^e^	5.31 ± 0.05 ^d^	nd	39.86 ± 0.59 ^d^	2.71 ± 0.01 ^f^
D2	0.36 ± 0.01 ^e^	4.51 ± 0.09 ^e^	nd	37.06 ± 0.18 ^ef^	2.83 ± 0.01 ^cd^
**Post-fermentation**					
A3	0.41 ± 0.01 ^e^	nd	nd	37.15 ± 0.09 ^ef^	2.88 ± 0.00 ^c^
B3	0.31 ± 0.01 ^e^	nd	nd	34.22 ± 0.55 ^g^	2.62 ± 0.02 ^g^
C3	0.32 ± 0.01 ^e^	1.13 ± 0.02 ^f^	nd	36.19 ± 0.32 ^f^	2.13 ± 0.11 ^h^
D3	0.39 ± 0.04 ^e^	nd	nd	44.24 ± 0.75 ^b^	3.01 ± 0.02 ^b^
**Maturation**					
A4	0.44 ± 0.04 ^e^	1.18± 0.05 ^f^	0.84 ± 0.06 ^e^	34.8± 0.22 ^g^	2.85 ± 0.03 ^cd^
B4	0.14 ± 0.03 ^e^	nd	nd	33.58 ± 0.08 ^g^	2.74± 0.00 ^ef^
C4	0.46 ± 0.02 ^e^	1.34 ± 0.14 ^f^	nd	36.82 ± 2.75 ^f^	2.80 ± 0.12 ^de^
D4	0.43 ± 0.04 ^e^	1.39 ± 0.04 ^f^	nd	42.56 ± 0.21 ^c^	3.03 ± 0.02 ^b^

Results are presented as a mean of three replicates ± standard deviation. The mean values presented in one column, marked with different letters (a, b, c, etc.) differ statistically (*p* < 0.05); nd – not detected.

**Table 3:** The content of xanthohumol (XN) and isoxanthohumol (IXN) in beers.

Sample	XN	IXN
	mg/L	
A4	1.93 ± 0.03 ^c^	0.96 ± 0.00 ^c^
B4	1.77 ± 0.02 ^d^	0.85 ± 0.00 ^d^
C4	2.55 ± 0.01 ^b^	1.03 ± 0.00 ^b^
D4	3.83 ± 0.05 ^a^	1.19 ± 0.01 ^a^

Results are presented as a mean of three replicates ± standard deviation. The mean values presented in one column, marked with different letters (a, b, c, etc.), differ statistically (*p* < 0.05).

**Table 4:** Color (EBC) and 5-hydroxymethylfurfural (5-HMF) content in worts and beers.

Sample	Color	5-HMF
	EBC	mg/L
Worts		
W0	106.73 ± 0.22 ^h^	18.92 ± 0.07 ^e^
W1	133.43 ± 3.08 ^b^	24.65 ± 0.03 ^b^
**Hopping**		
A1	114.15 ± 0.00 ^fg^	20.05 ± 0.46 ^d^
B1	139.58 ± 2.32 ^a^	23.4 ± 0.29 ^c^
C1	142.95 ± 0.45 ^a^	26.52 ± 0.01 ^a^
D1	141.60 ± 0.90 ^a^	26.72 ± 0.12 ^a^
**Main fermentation**		
A2	107.10 ± 2.10 ^h^	nd
B2	113.03 ± 0.08 ^fg^	nd
C2	126.45 ± 3.30 ^cd^	nd
D2	132.08 ± 1.58 ^b^	nd
**Post-fermentation**		
A3	122.93 ± 2.03 ^de^	nd
B3	114.68 ± 0.53 ^f^	nd
C3	131.03 ± 2.32 ^b^	nd
D3	131.40 ± 1.80 ^b^	nd
**Maturation**		
A4	121.32 ± 3.30 ^e^	nd
B4	110.40 ± 4.95 ^gh^	nd
C3	123.15 ± 2.85 ^de^	nd
D4	130.05 ± 0.30 ^bc^	nd

Results are presented as a mean of three replicates ± standard deviation. The mean values presented in one column, marked with different letters (a, b, c, etc.) differ statistically (*p* < 0.05); nd – not detected.

**Table 5:** The total content of polyphenolic compounds (TPC), the ability to reduce iron ions (FRAP), and the antioxidant capacity (ABTS^•+^) of wort and beers at subsequent technological stages.

Sample	TPC	FRAP	ABTS^+·^
	mg GAE/L	mmol TE/L	mmol TE/L
Worts			
W0	336.13 ± 3.92 ^k^	1.61 ± 0.05 ^l^	0.91 ± 0.01 ^g^
W1	388.19 ± 10.67 ^j^	1.94 ± 0.03 ^k^	1.27 ± 0.09 ^ef^
**Hopping**			
A1	418.00 ± 16.57 ^i^	2.56 ± 0.04 ^i^	1.53 ± 0.1 ^cd^
B1	387.53 ± 10.03 ^j^	2.32 ± 0.06 ^j^	1.61 ± 0.16 ^bc^
C1	383.82 ± 15.93 ^j^	2.71 ± 0.07 ^gh^	1.75 ± 0.00 ^ab^
D1	477.1 ± 20.56 ^h^	2.78 ± 0.03 ^fg^	1.76 ± 0.06 ^ab^
**Main fermentation**			
A2	676.74 ± 6.51 ^ef^	2.9 ± 0.17 ^e^	1.51 ± 0.24 ^cd^
B2	670.2 ± 14.08 ^f^	2.39 ± 0.09 ^j^	1.21 ± 0.13 ^f^
C2	713.8 ± 9.12 ^cd^	2.85 ± 0.01 ^ef^	1.43 ± 0.05 ^cde^
D2	623.91 ± 9.35 ^g^	2.78 ± 0.07 ^fg^	1.91 ± 0.02 ^a^
**Post-fermentation**			
A3	693.34 ± 17.01 ^de^	2.67 ± 0.03 ^gh^	1.38 ± 0.04 ^def^
B3	740.1 ± 5.44 ^b^	2.61 ± 0.03 ^hi^	1.26 ± 0.06 ^ef^
C3	840.75 ± 14.89 ^a^	2.36 ± 0.03 ^j^	1.49 ± 0.1 ^cd^
D3	732.04 ± 8.29 ^bc^	3.08 ± 0.03 ^d^	1.43 ± 0.08 ^cde^
**Maturation**			
A4	433.32 ± 9.54 ^i^	3.64 ± 0.05 ^c^	1.18 ± 0.1 ^f^
B4	418.32 ± 11.63 ^i^	4.88 ± 0.04 ^a^	1.2 ± 0.11 ^f^
C4	371.51 ± 10.94 ^j^	3.73 ± 0.05 ^c^	1.24 ± 0.21 ^ef^
D4	475.05 ± 23.30 ^h^	3.86 ± 0.04 ^b^	1.43 ± 0.11 ^cde^

Results are presented as a mean of three replicates ± standard deviation. The mean values presented in one column, marked with different letters (a, b, c, etc.) differ statistically (*p* <0.05).

## Data Availability

The data presented in this study are available on request from the corresponding author.
